# Diabetes, Obesity, and Osteoporosis in an Ethnically Diverse Population of Women Receiving Osteoporosis Screening

**DOI:** 10.1093/geroni/igab046.3480

**Published:** 2021-12-17

**Authors:** Feven Kahsay, Wendy Yang, Malini Chandra, Catherine Lee, Nailah Thompson, Joan Lo

**Affiliations:** 1 McGovern Medical School, Houston, Texas, United States; 2 The Permanente Medical Group, San Jose, California, United States; 3 Kaiser Permanente Northern California, Oakland, California, United States; 4 The Permanente Medical Group, Oakland, California, United States

## Abstract

Osteoporosis screening by bone density (BMD) testing is recommended for women aged 65-75 years. However, patients with diabetes, a risk factor for fracture, often have higher body mass index (BMI) which contributes to higher BMD. These factors may vary by race/ethnicity. The relationship of diabetes (≥2 diagnoses and treatment), obesity (BMI ≥30), and BMD-defined osteoporosis (femoral neck BMD T-score ≤ -2.5) was examined in a diverse primary care population of 44,313 non-Hispanic White, 6,103 Black, 7,777 Hispanic, and 12,634 Asian women aged 65-75 years who underwent BMD screening. Those with recent fracture, osteoporosis treatment, bone disorders, and metastatic cancer were excluded. Modified log-Poisson regression was used to examine the association of diabetes and BMD-osteoporosis. Among 70,827 women, 18% had diabetes. The prevalence of diabetes was 2-fold higher in Black, Hispanic and Asian women compared to White women. Overall, women with diabetes (versus no diabetes) were more likely to be obese and, except for Hispanic women, less likely to have BMD-osteoporosis. In unadjusted analyses, diabetes was associated with lower risk of BMD-defined osteoporosis in White, Black, and Asian women, but not Hispanic women. However, the association was attenuated or no longer evident after adjusting for BMI, suggesting that the lower burden of BMD-osteoporosis in women with diabetes is mediated in part by higher BMI. These findings support consideration of diabetes when assessing fracture risk in women undergoing osteoporosis screening. However, more studies in non-White populations with a high burden of diabetes are important since these relationships appear to differ by race/ethnicity.

